# ^18^F-labeled tracers targeting fibroblast activation protein

**DOI:** 10.1186/s41181-021-00144-x

**Published:** 2021-08-21

**Authors:** Thomas Lindner, Annette Altmann, Frederik Giesel, Clemens Kratochwil, Christian Kleist, Susanne Krämer, Walter Mier, Jens Cardinale, Hans-Ulrich Kauczor, Dirk Jäger, Jürgen Debus, Uwe Haberkorn

**Affiliations:** 1 Department of Nuclear Medicine, Heidelberg University Hospital, Im Neuenheimer Feld 400, 69120 Heidelberg, Germany; 2 Clinical Cooperation Unit Nuclear Medicine, German Cancer Research Center (DKFZ), Heidelberg, Germany; 3 Department of Radiology, Heidelberg University Hospital, Heidelberg, Germany; 4 Translational Lung Research Center Heidelberg (TLRC), German Center for Lung Research (DZL), Heidelberg, Germany; 5 Department of Medical Oncology, National Center for Tumor Diseases (NCT), Heidelberg, Germany; 6 Department of Radiation Oncology, Heidelberg University Hospital, Heidelberg, Germany; 7 Clinical Cooperation Unit Radiation Oncology, German Cancer Research Center (DKFZ), Heidelberg, Germany

**Keywords:** Fibroblast activating protein, Positron Emission Tomography, 18F-AlF-FAPI-74

## Abstract

**Background:**

Cancer-associated fibroblasts are found in the stroma of epithelial tumors. They are characterized by overexpression of the fibroblast activation protein (FAP), a serine protease which was already proven as attractive target for chelator-based theranostics. Unfortunately, the value of gallium-68 labeled tracers is limited by their batch size and the short nuclide half-life. To overcome this drawback, radiolabeling with aluminum fluoride complexes and 6-fluoronicotinamide derivatives of the longer-lived nuclide fluorine-18 was established. The novel compounds were tested for their FAP-specific binding affinity. Uptake and binding competition were studied in vitro using FAP expressing HT-1080 cells. HEK cells transfected with the closely related dipeptidyl peptidase-4 (HEK-CD26) were used as negative control. Small animal positron emission tomography imaging and biodistribution experiments were performed in HT-1080-FAP xenografted nude mice. [^18^F]AlF-FAPI-74 was selected for PET/CT imaging in a non-small cell lung cancer (NSCLC) patient.

**Results:**

In vitro, ^18^F-labeled FAPI-derivatives demonstrated high affinity (EC_50_ = < 1 nm to 4.2 nm) and binding of up to 80% to the FAP-expressing HT1080 cells while no binding to HEK-CD26 cells was observed. While small animal PET imaging revealed unfavorable biliary excretion of most of the ^18^F-labeled compounds, the NOTA bearing compounds [^18^F]AlF-FAPI-74 and -75 achieved good tumor-to-background ratios, as a result of their preferred renal excretion. These two compounds showed the highest tumor accumulation in PET imaging. The organ distribution values of [^18^F]AlF-FAPI-74 were in accordance with the small animal PET imaging results. Due to its less complex synthesis, fast clearance and low background values, [^18^F]AlF-FAPI-74 was chosen for clinical imaging. PET/CT of a patient with metastasized non-small cell lung cancer (NSCLC), enabled visualization of the primary tumor and its metastases at the hepatic portal and in several bones. This was accompanied by a rapid clearance from the blood pool and low background in healthy organs.

**Conclusion:**

[^18^F]AlF-labeled FAPI derivatives represent powerful tracers for PET. Owing to an excellent performance in PET imaging, FAPI-74 can be regarded as a promising precursor for [^18^F]AlF-based FAP-imaging.

**Supplementary Information:**

The online version contains supplementary material available at 10.1186/s41181-021-00144-x.

## Background

Cancer associated fibroblasts (CAFs) are an essential component of the tumor stroma in the vast majority of epithelial cancers and are known to contribute to tumor growth, migration and progression (Gascard and Tlsty 2016; Lamprecht et al. 2018). A key characteristic of most CAFs is the expression of the fibroblast activation protein (FAP), a membrane bound serine protease belonging to the dipeptidyl peptidase 4 (DPP4) family. FAP is involved in a variety of tumor promoting activities such as matrix remodeling, angiogenesis, chemotherapy resistance and immunosuppression. Therefore, it represents a potent biomarker for cancer prognosis. Due to its overexpression in the tumor environment, combined with low expression in most normal tissues, FAP is considered as an important target for diagnostic imaging and anti-cancer therapies in nuclear medicine (Gascard and Tlsty 2016; Lamprecht et al. 2018; Lindner et al. 2019; Marsh et al. 2013; Plava et al. 2019; Pure and Lo 2016; Calais 2020).

Based on specific inhibitors of the serine protease (Jansen et al. 2013, 2014), FAP targeting small molecule radiotracers (FAPI) have been developed recently (Lindner et al. 2018; Loktev et al. 2018). By chelation of various radiometals, these radiopharmaceuticals showed high affinity binding and rapid internalization into FAP-expressing tumor cells in radioligand assays (Lindner et al. 2018, 2020; Loktev et al. 2018, 2019). By means of ^68^ Ga-labeling, positron-emission-tomography (PET) allowed imaging of FAP-expressing tumor lesions in animal models with high tumor-to-background ratios. An important advantage of the FAPI-tracers is their ability to detect malignancies with low glucose uptake. This improves imaging in patients suffering from tumors such as NSCLC, which are not readily detectable by FDG-PET/CT. Notably, a first comparison with the commonly used radiotracer ^18^F‐FDG revealed a superiority of ^68^ Ga-FAPI-04 in patients with different tumor entities (Giesel et al. 2019a, b,^ 2021^; Kratochwil et al. 2019; ·Li ·et al. 2021; Kuten et al. 2021).

While the DOTA-chelator present in the radiotracers enables a therapeutic option, the dependence on ^68^ Ga for PET imaging poses a drawback to a broader application. One main reason is the typical maximal batch size of 2–4 GBq for ^68^Ge/^68^ Ga generators which decreases with the half-life of ^68^Ge (271 days). Another reason is the short half-life of ^68^ Ga (68 min). This issue requires an on-site and on-time synthesis of the radiotracer. Accordingly, the application of ^18^F-labeled compounds as PET tracer provides an attractive alternative for diagnostic imaging (Cardinale et al. 2017; Giesel et al. 2019c; Richter and Wuest 2014). Although its production requires a medical cyclotron and a properly shielded facility, the half-life of ^18^F and batch sizes of several hundred GBq enable the delivery of tracers to remote PET centers without radiopharmaceutical department. Consequently, we aimed towards the development of a ^18^F-labeled FAP tracer.

As a first step, a suitable labeling strategy had to be selected, in particular since the performance of the FAPI-radiotracers is strongly affected by molecule size as well as lipophilicity (Lindner et al. 2018, 2020; Loktev et al. 2018, 2019). Furthermore, in comparison to chelation of radiometals, classic ^18^F-substitution reactions usually require large amounts of precursor, multi-step syntheses and harsh reaction conditions. In addition to the chelation with [^18^F]aluminum fluoride, the substitution at 6-trimethylammoniumnicotine derivatives was chosen for its relative low precursor consumption, polarity of the prosthetic group and one-step radiolabeling (Richter and Wuest 2014; Jacobson et al. 2015; Basuli et al. 2016; McBride et al. 2009; Olberg et al. 2010; Richarz et al. 2014). In order to obtain precursors for fluorination, FAPI-tracers containing 6-flouronicotineamides and aluminum fluoride-NOTA complexes were designed.

Herein, we describe the synthesis and the in vitro and in vivo evaluation of ^18^F-labeled FAPI derivatives. The clinical value of one of these compounds was proven by the PET-imaging of a patient with NSCLC.

## Materials and methods

### Compound synthesis

All solvents and non-radioactive reagents were obtained in reagent grade from ABCR (Karlsruhe, Germany), Sigma-Aldrich (München, Germany), Acros Organics (Geel, Belgium) or VWR (Bruchsal, Germany) and were used without further purification. Precursors carrying the chelating moiety NOTA were synthesized similarly to previously described procedures (Lindner et al. 2018; Loktev et al. 2018). 2,3,5,6-Tetrafluorophenlyl 6-trimethylaminiumnicotinate chloride salt was synthesized according to Olberg et al. (Olberg et al. 2010), using a 2 m solution of trimethylamine in tenfold excess instead of the passing through of trimethylamine gas. Further details and additional protocols are given in the supporting information.

### Analysis

HPLC analyses were performed on Agilent 1100 systems with VWD-detectors (Agilent Technologies Germany) equipped with Chromolith Performance RP18e columns (3 × 100 mm; Merck, Germany). UV-traces were recorded at 214 nm using the included ChemStation software. Solvents used were water and acetonitrile, each containing 0.1% trifluoroacetic acid. Unless noted otherwise a linear gradient of 0–100% acetonitrile in 5 min was chosen. In case of radioactive compounds, an equal setup equipped with a gamma detector (GABI, Elysia-Raytest, Germany) was used.

### Radiolabeling

Radiolabeling of 6-trimethylammonium nicotinamides FAPI-72 and -73 was performed by the previously established methods of Richarz et al. (2014) and Basuli et al. (2016). Briefly, 1–4 GBq [^18^F]fluoride (ZAG Zykloron AG, Karlsruhe, Germany) in 2 mL water were trapped on an anion exchange cartridge (Waters Accel Plus QMA Light cartridge), preconditioned with 5 mL 1 m KHCO_3_ and 10 mL of water, washed with 3 mL acetonitrile and dried by a stream of nitrogen. Elution was performed with 0.45–0.55 mg of the individual precursor dissolved in 500 µL ethanol. After evaporation of the solvent under reduced pressure, 100 µL tert-butanol/acetonitrile 4:1 were used to dissolve the residue. The mixture was heated at 70 °C for 15 min before fractions containing 100–200 MBq of the reaction mixture were diluted with 150 µL water/acetonitrile 3:1 and purified by preparative HPLC (LaChrom L7100, Merck, Darmstadt, Germany; Chromolith Performance RP18e 100 × 4.6 mm, Merck, Darmstadt, Germany; 0–30% acetonitrile in 10 min). Subsequently, the solvents were removed and the residue dissolved in 0.9% NaCl for animal studies.

Chelations of [^18^F]AlF for FAPI-42, -52 and -74 to -76 were performed according to the protocol of McBride et al. (2009). 2–10 GBq [^18^F]fluoride (ZAG Zyklotron AG, Karlsruhe, Germany) in 4 mL water were trapped on an anion exchange cartridge (Waters Accel Plus QMA Light cartridge, preconditioned with 5 mL 0.5 m NaOAc pH 3.9 and 10 mL of water) and eluted with 0.3 mL 0.5 m NaOAc pH 3.9. The solution was incubated with 6 µL of AlCl_3_ in water (10 mm) and 300 µL DMSO for 5 min at room temperature before 20 µL of the respective precursor (4 mm) was added. The reaction was carried out at 95 °C for 15 min, cooled to room temperature, diluted with 5 mL water and worked up by SPE (Waters Oasis HLB Plus Light cartridge). Subsequently, the solvents (1 mL of water/ethanol 1:1) were removed and the residue dissolved in 0.9% NaCl for animal studies.

In case of clinical application, the product was eluted (1 mL of water/ethanol 1:1) into a sterile 20 mL vial with the use of a sterile syringe filter. Subsequently, the filter was rinsed with 10 mL sterile 0.9% saline and 0.5 mL sterile phosphate buffer to dilute the preparation to less than 5% ethanol content. Finally, a reference sample was drawn from the final product, which was analyzed by means of radio-HPLC (product-peak area higher than 95%) and tested for neutral pH (pH 6–8).

### Determination of logD values

All experiments for the determination of logD values were performed in triplicate. For the determination by means of radioactivity, around 5 × 10^6^ cpm in ca. 1 µL of radioactive stock solutions (water/acetonitrile 1:1) were added to 100 µL phosphate buffered saline (PBS, pH 7.4) and 100 µL of 1-octanol. The mixture was vortexed for 1 min, followed by 5 min of centrifugation for phase separation. A sample of each phase (10 or 50 µL) was measured for radioactivity per volume (Packard Cobra II Autogamma, GMI Inc., USA). In case of FAPI-72 and -73, the reference compounds [^19^F]-FAPI-72 and -73 were dissolved in PBS at a concentration of 1 mg/mL. 50 µL of these solutions were each mixed with 50 µL 1-octanol and processed by vortexing and centrifugation as described above. 1 µL of each phase was analyzed by HPLC and the individual content of compound was determined by peak integration. The area under the peaks of the individual runs was used to calculate the log D value.

### Serum stability assay

Five MBq of purified [^18^F]AlF-FAPI-74 (20 GBq/µmol) were dried under reduced pressure and the residue incubated in 300 µL human serum (Sigma Aldrich Germany) at 37 °C. After different time intervals (10 min to 4 h) 20 µL samples were precipitated with 40 µL acetonitrile. The stability of the labeled peptide was monitored by radio-HPLC of the supernatant. A gradient of 0% to 30% acetonitrile in 10 min was used for enhanced separation performance.

### In vitro binding experiments

The binding properties of the FAPI derivatives were evaluated using the FAP‐transfected HT‐1080‐FAP and CD26 expressing HEK-CD26 cells. All cells were cultivated in Dulbecco's modified Eagle's medium (DMEM) containing 10% fetal calf serum (FCS) at 37 °C/5% carbon dioxide. Cells were seeded in 6-well plates and cultivated to a final confluence of approximately 80–90% (1.2 to 2.0 × 10^6^ cells/well). After washing with FCS free medium, for each well ca. 3 kBq of the radiolabeled compound (10–20 GBq/µmol for [^18^F]AlF-labeling) was added to the cells in FCS free medium. On completion of the individual incubation time, cells were washed with PBS and treated with 1.4 mL lysis buffer (0.3 m NaOH, 0.2% SDS) before being transferred to measuring tubes. Competition experiments were performed by co-incubation with the unlabeled precursor for 60 min. Radioactivity was determined in a Wizard Gamma Counter (Perkin Elmer), normalized to 10^6^ cells and calculated as percentage of the applied dose (%AD). Each experiment was performed in triplicate.

### Animal studies

Eight-week-old BALB/c nu/nu mice (Charles River) were subcutaneously inoculated with 5 × 10^6^ HT-1080-FAP cells. The subcutaneous xenografts were grown at the flanks of the mice. Xenografts were grown to a tumor diameter of 10–15 mm. Mice were anesthetized using isoflurane inhalation. 5–10 MBq of the [^18^F]AlF-labeled compounds/ the respective tracer (FAPI-42/52/74/75/76; approx. 0.5 nmol; A_m_ approximately 10–20 GBq/µmol) were injected intravenously for PET imaging. In vivo competition (blocking) experiments with ^68^ Ga-FAPI-74 (9 MBq; 18.2 GBq/µmol) were performed by adding 30 nmol of unlabeled FAPI-74 to the injection solution. Images were acquired using a small-animal PET scanner (Inveon, Siemens). Within the first 60 min p.i., a dynamic scan was performed in list mode, followed by a static scan from 120–140 min post injection. Images were reconstructed iteratively using the 3D-OSEM + MAP method (Siemens) and were converted to standardized uptake value (SUV). For the dynamic analysis, 28 frames were reconstructed. Quantification was based on ROI analysis and expressed as SUV.

To reduce radiation exposure, the organ distribution study was performed with 1 MBq per animal. The specific activity was lowered to provide the amount of precursor injected per animal being equal to the small animal PET experiments (approximately 0.5 nmol; A_m_ approximately 2 GBq/µmol). The animals were sacrificed 30, 60, 120 and 240 min p.i., organs of interest dissected and weighted. The radioactivity was measured using a γ-counter (Packard Cobra II Autogamma, GMI Inc., USA) and expressed as percentage of injected dose per gram of tissue (%ID/g). PET imaging of [^18^F]AlF-FAPI-74 and -75 as well as the biodistribution were performed in triplicate.

### Clinical PET/CT-imaging

[^18^F]AlF-FAPI-74 (16.2 GBq/µmol at time of injection) was applied intravenously (20 nmol; 323 MBq) to a 68 y old patient with NSCLC. The PET/CT scans were performed 1 and 3 h post tracer administration with a Biograph mCT Flow™ PET/CT-Scanner (Siemens Medical Solutions). The parameters used were slice thickness of 5 mm, increment of 3–4 mm, soft-tissue reconstruction kernel and care dose. After CT scanning, a whole-body PET was acquired in 3D (matrix 200 × 200) in FlowMotion™ with 0.7 cm/min. The emission data were corrected for random, scatter and decay. Reconstruction was conducted with an ordered subset expectation maximization (OSEM) algorithm with 2 iterations/21 subsets and Gauss-filtered to a transaxial resolution of 5 mm at full-width half-maximum. Attenuation correction was performed using the low-dose non-enhanced CT data. The quantitative assessment of standardized uptake values (SUV) was done using a region of interest technique.

## Results

### Synthesis of precursors and ^18^F-labeled FAPI-tracers

**Fig. 1:**
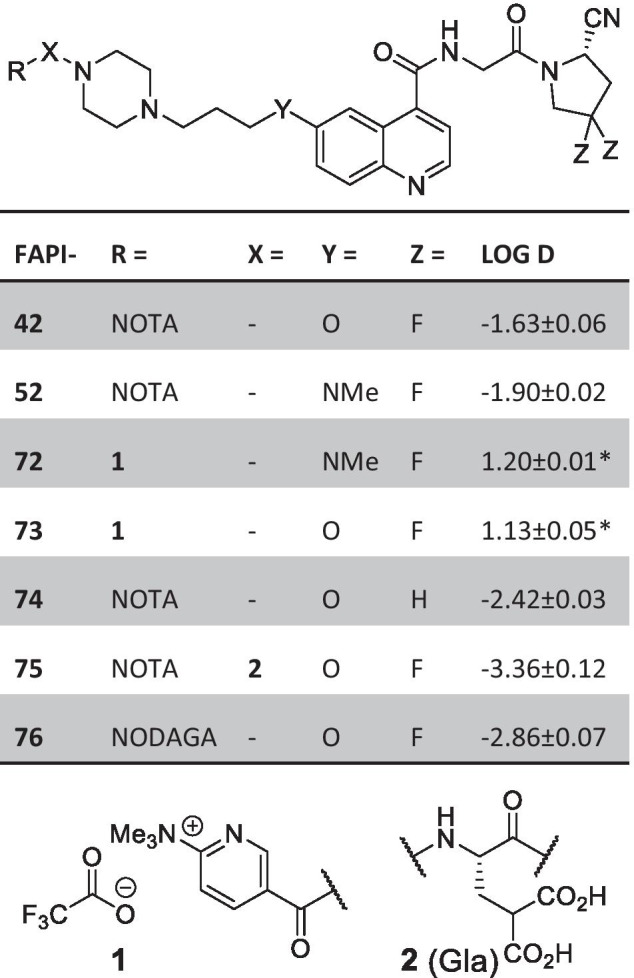
Overview of the synthesized precursors discussed in this study. The corresponding log D values were determined by detection of the radiolabeled tracer in 1-octanol and phosphate buffered saline (pH 7). For values labeled with an asterix, the log D was calculated from the relative content of the non-radioactive reference standards in the 1-octanol and the water phase by HPLC analysis and peak integration

A total of 7 different precursors for radiofluorination were synthesized (Fig. 1). The 1-step coupling of the activated esters for all tracers except FAPI-75 proceeded with 70–94% yield. The 1-step coupling / Fmoc-deprotection of l-(γ,γ’-bis-*tert*-butyl)carboxyglutamic acid provided 83%, the following active ester coupling and *tert*-butyl deprotection 63% yield over two steps. Comprehensive details of the syntheses, compound characterization and ^18^F-fluorination are provided in the supporting information.

For the radiofluorination of the 6-fluoronicotineamides (FAPI-72 and -73), the precursor (approx. 0.5 mg) dissolved in ethanol was used to elute the radionuclide. This change in solvent was necessary since, although methanol proofed more efficient for [^18^F]fluoride elution, a hardly removable methoxy-substituted byproduct was formed in preliminary tests with ^19^F. Labeling reactions showed complete conversion in HPLC and the products could be isolated in 50–70% RCY after HPLC-purification.

For the formation of the [^18^F]AlF-NOTA complexes, a 0.5 m sodium acetate buffer at pH 3.9 was used and the products were obtained in labeling yields of 40–70% and non-decay corrected yields of 15–30%.

A stability test of [^18^F]AlF-FAPI-74 was conducted in human serum and showed no degradation of the tracer or instability of the [^18^F]AlF-NOTA complex over the course of 4 h (Additional file 1: Fig. S2).

### Uptake experiments of ^18^F -FAPI derivatives with recombinant FAP-expressing and control cell lines

Table 1 summarizes the uptake and EC_50_ values of the discussed ^18^F-labeled FAPI derivatives using the FAP-transfected cell line HT-1080-FAP. Additionally, the lead candidates FAPI-74 to -76 were tested on HEK-CD26 as well as -74 and -75 on wild type HT1080 cells as negative control. Except for FAPI-74, all compounds showed a strong increase in binding between 10 and 60 min. No significant improvement was observed at later time points. For FAPI-74, a plateau was already reached after approximately 10 min with 27.2 ± 0.88%AD/10^6^cells. Later time points showed minor increase in binding. The EC_50_ values were all in the low to sub-nanomolar range. Only a neglectable fraction of FAPIs 74–76 were detected on HEK-CD26 cells (0.18–0.24%) as well as on wildtype HT1080 in case of -74 and 75 (< 0.1%).

**Table 1:** Uptake experiments of ^18^F-labeled FAPI-derivatives with HT1080-FAP and control cell lines

FAPI-	10 min	60 min	120 min	CD26	WT	EC50 [nm]
42	40.0 ± 1.73	61.2 ± 1.76	58.0 ± 2.24	n.d	n.d	3.3
52	32.0 ± 0.28	45.9 ± 0.54	53.2 ± 1.38	n.d	n.d	3.2
72	13.9 ± 0.60	23.6 ± 0.40	24.4 ± 0.30^a^	n.d	n.d	< 1^b^
73	22.7 ± 0.24	39.2 ± 0.48	39.6 ± 0.95^a^	n.d	n.d	< 1^b^
74	27.2 ± 0.88	29.7 ± 0.66	30.39 ± 2.47	0.18 ± 0.04	0.07 ± 0.03	4.2
75	34.9 ± 0.26	74.0 ± 2.07	82.35 ± 3.61	0.18 ± 0.02	0.06 ± 0.01	3.1
76	45.1 ± 2.20	74.9 ± 0.82	73.05 ± 0.99	n.d	n.d	1.6

All values except EC_50_ values are given in percent of applied dose on 1 million cells (%AD/10^6^ cells) with standard deviations. ^a^Measurement at 90 min. ^b^Out of confidence range

### In Vivo targeting properties of ^18^F-labeled FAPI derivatives

**Fig. 2:**
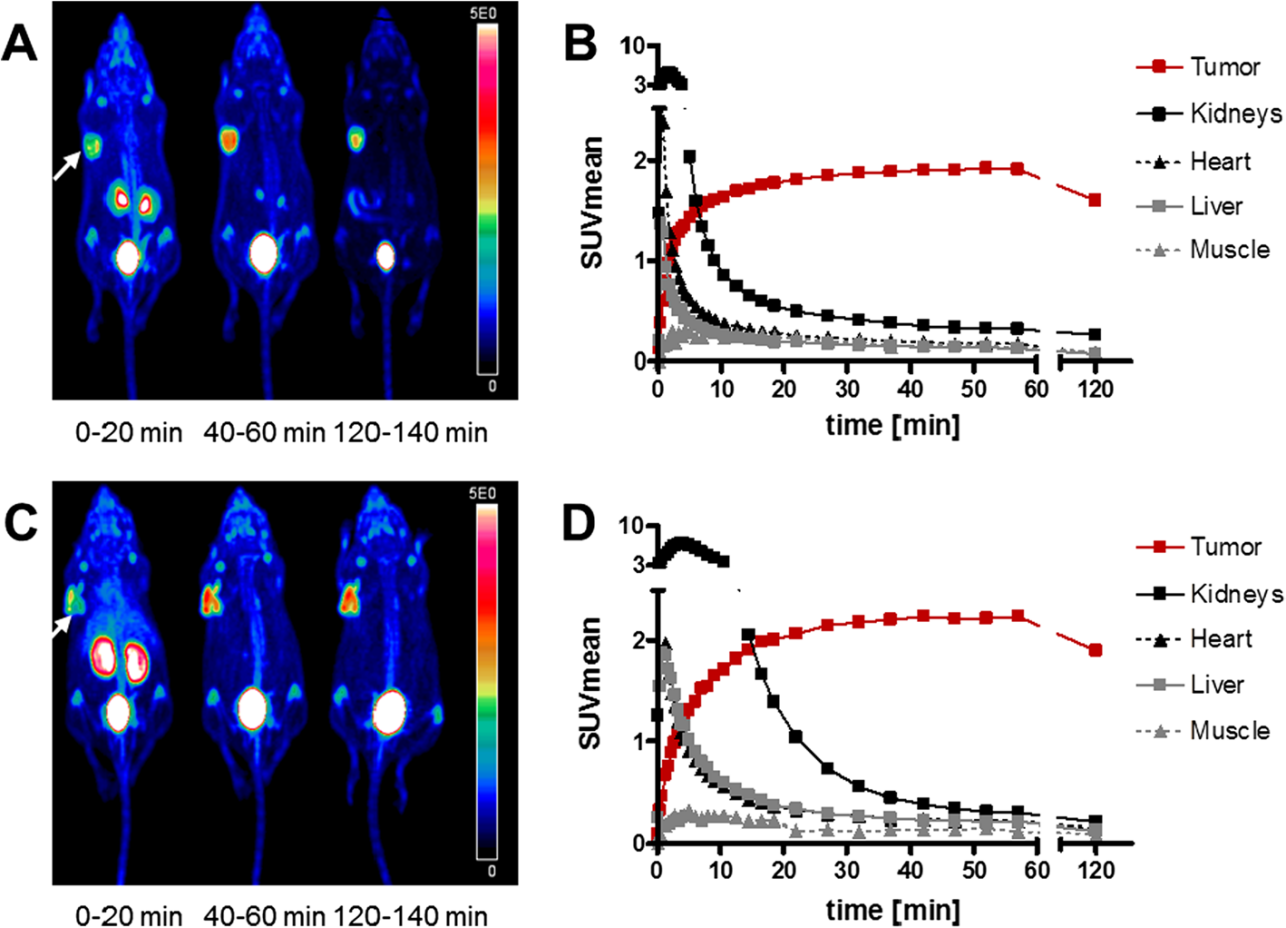
Small animal PET imaging. The white arrow indicates the site of the xenotransplanted tumor. Shown are the maximum intensity projections (**A**, **C**) and time-activity curves (**B**, **D**) of selected organs in case of [^18^F]AlF-FAPI-74 (**A**, **B**) and [^18^F]AlF-FAPI-75 (**C**, **D**). Experiments were conducted in nude mice transplanted with HT-1080-FAP cells and images were acquired in the indicated time periods after tracer administration

Small animal PET studies with [^18^F]AlF-FAPI-74 and -75 showed a fast clearance and tumor uptake with only trace amounts of activity found in the intestine (Fig. 2). While [^18^F]AlF-FAPI-75 showed slightly higher tumor-SUVs (3.7 vs. 2.3 max; 2.2 vs. 1.9 mean), [^18^F]AlF-FAPI-74 images offered better tumor to background ratios (Fig. 2). All other compounds revealed an unfavorable excretion via the hepatobiliary pathway (Additional file 1: Fig. S3). In case of [^18^F]-FAPI-72 and -73, the tumor region was hardly detectable, whereas high activity was detected in the intestine and bladder. A list of the tumor uptakes (SUVmean values) for all tested compounds at different time points between 0 and 120 min can be found in Additional file 1: Table S1.

**Fig. 3:**
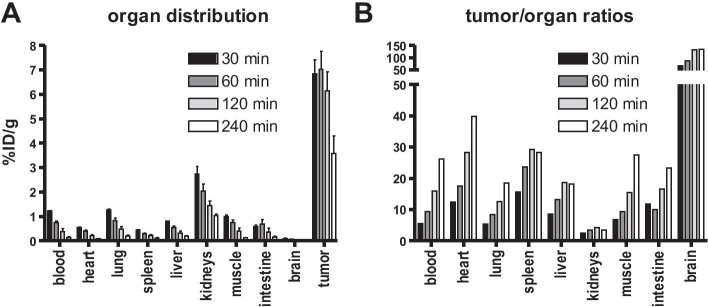
Biodistribution study of [^18^F]AlF-FAPI-74. Accumulation in different tissues (**A**) and tumor-to-organ ratios (**B**) in HT-1080-FAP bearing Balb/c nu/nu mice 30, 60, 120, and 240 min after injection

In accordance with the PET results, the biodistribution study of [^18^F]AlF-FAPI-74 (Fig. 3) confirmed a good/high tumor uptake of 6.9%ID/g after 30 min, which was stable until 1 h p.i. and then started to decrease slowly. The compound showed a good clearance from all non-target organs resulting in very good tumor to background ratios.

### FAPI-74 accumulates in NSCLC

**Fig. 4:**
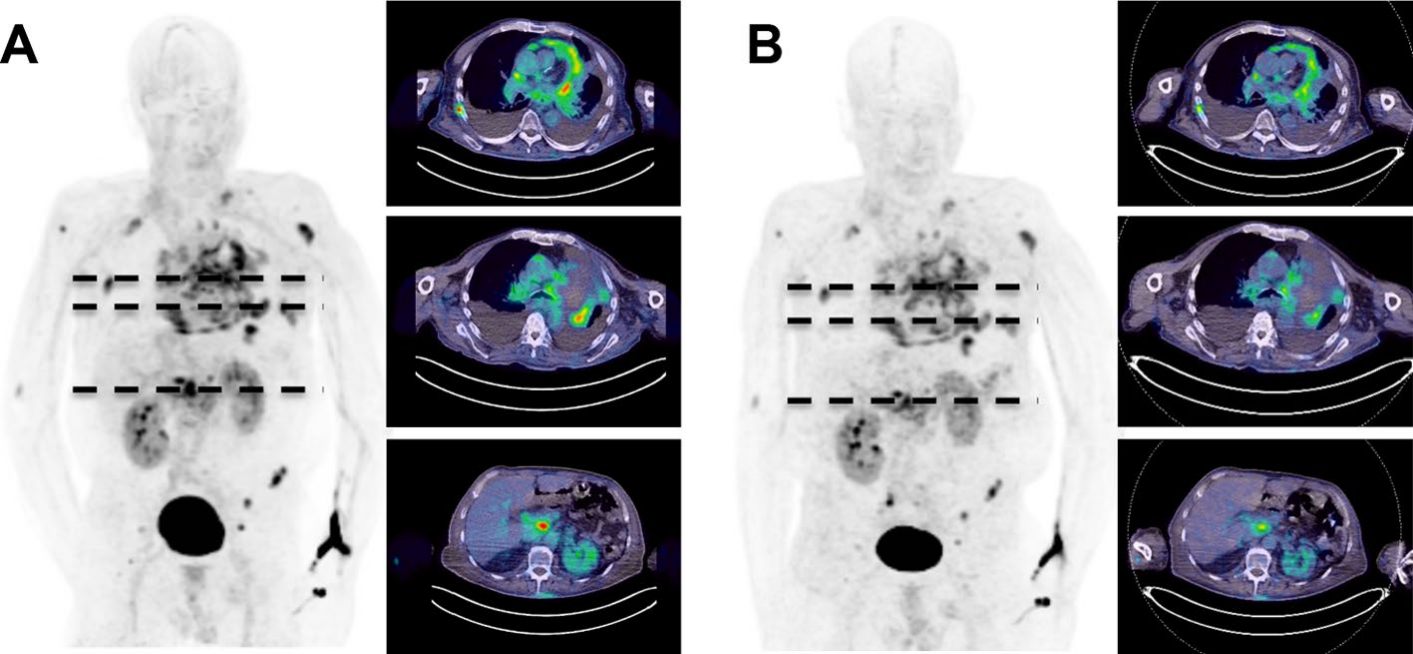
Whole body PET/CT images of a 68y old patient with NSCLC in the left upper lobe. Shown are the maximum intensity projections and transaxial cuts at **A** 1 h and **B** 3 h after administration of 323 MBq [^18^F]AlF-FAPI-74

Finally, [^18^F]AlF‐FAPI‐74 was used for PET/CT imaging in a 68y old patient with metastasized NSCLC prior to radiation therapy. Whole body PET/CT scans were performed 1 and 3 h after intravenous administration of the radiotracer. Rapid accumulation of activity in the primary tumor and in metastases at the hepatic portal and in several bones (n = 7) was observed. Maximum SUV (SUVmax) values of 6.5 ± 1.1 (1 h) and 5.6 ± 0.7 (3 h), respectively (SUVmean at 1 h was 3.3 ± 0.6 and at 3 h 2.75 ± 0.5), but almost zero in normal tissues were detected (Fig. 4). Tracer uptake in multiple nodules in the pericardium was considered as evidence for a pericardial infiltration. Radioactivity in non-target tissues and the blood pool was rapidly cleared and excreted predominantly via the kidneys, resulting in images with high contrast.

## Discussion

Radiolabeling of ligands with ^18^F offers advantages over ^68^ Ga-labeled ligands such as the possibility of the production of large batches and the longer half-life of the nuclide. This allows the increase of the number of patients and the possibility of tracer distribution from production sites to remote imaging centers (satellite concept). In this concept only the production site has to install and maintain the cost-intensive cyclotron facility, while remote PET centers could directly obtain a GMP compliant product without the need for on-site production. Moreover, the lower energy of the positron particle emitted by ^18^F may lead to an improved detection of smaller lesions due to a lower partial volume effect. While chelations with ^68^ Ga are single step reactions which usually require 20 µg per synthesis, substitutions with ^18^F require up to multiple milligrams and additional steps if protecting groups have to be removed. By reason of mild reaction conditions and low precursor consumption, the radiolabeling of 6-trimethylammoniumnicotinamides and chelation of an aluminum fluoride complex were chosen for this work. Several FAPI variants including [^18^F]6-fluoronicotinamides and [^18^F]AlF-complexes were designed and evaluated in in vitro and in vivo (Additional file 2).

All variants revealed EC_50_ values in the low- or sub-nanomolar range and favorable binding characteristics. So far, FAPI-75 and -76, compounds with additional negatively charged carboxylate(s) achieved the highest accumulation in vitro. For the lead candidates FAPI-74–76 (for selection see below) further in vitro control experiments with the HEK-CD26 cells and the wildtype HT1080 as well as the in vivo blocking experiments confirmed the high specificity of the tracers. Additionally, it should be noted that the specificity of the FAPI scaffold was already known from previous in vivo studies using [^68^ Ga]Ga-FAPI-04/46 (Lindner et al. 2018; Loktev et al. 2019).

For the selection of a lead candidate for further evaluation, all compounds were screened in vivo by small animal PET in tumor bearing mice. The lead candidates [^18^F]FAPI-74 and -75 were selected based on their favorable renal excretion in combination with a good tumor uptake of approx. 2.0 SUV_mean_ for both compounds. All other compounds showed an unfavorable hepatobiliary excretion. However, it should be noted that [^18^F]FAPI-42 at least showed an acceptable tumor uptake of approx. 1.0 SUV_mean_ at early timepoints (Additional file 3).

Small animal PET imaging with [^18^F]AlF-FAPI-74 and -75 further revealed a good tumor accumulation and contrast, both comparable to ^68^ Ga-FAPI-04. As a result of the higher affinities caused by the difluoro substitution at the cyanopyrrolidine, a higher tumor uptake was observed for [^18^F]AlF-FAPI-75. By contrast, the time activity curves showed a lower uptake in non-target tissues for [^18^F]AlF-FAPI-74, also accompanied by a faster clearance from these organs. The focus of these considerations was the timeframe of the accumulation in the heart as a surrogate for blood activity, which were clearly in favor of [^18^F]AlF-FAPI-74. This also leads to a lower radiation burden to the sensitive bone marrow. Therefore, FAPI-74 was chosen for the application in patients to minimize radiation exposure risks.

The promising results from small animal PET were further confirmed by ex vivo biodistribution experiments which were only performed for this tracer for reasons of animal welfare. [^18^F]AlF-FAPI-74 showed an early and good tumor uptake of approximately 7%ID/g. At later timepoints the tracer showed a slight washout. However, the quick clearance from non-target tissues was also confirmed in biodistribution qualifying the tracer as an excellent candidate for translation into a clinical setting.

[^18^F]AlF-FAPI-74 was applied in a compassionate use setting in a patient with NSCLC prior to a possible radiation therapy. The images taken at 1 h and 3 h p.i. showed an imaging performance equal to ^68^ Ga labeled FAPIs in previous reports (Lindner et al. 2018; Loktev et al. 2018, 2019; Giesel et al. 2019a; Kratochwil et al. 2019). The quantitative analysis at 1 h and 3 h revealed a decrease in SUV from 1 to 3 h, which is also reported in a larger series of patients with lung cancer. Measurement at 10 min, 1 h and 3 h revealed a maximum value at 1 h after injection (Giesel et al. 2020).

Recently, Toms et al*.* published a [^18^F]-6-fluoro-6-deoxyglycosylated FAP derivative (Toms et al. 2020). Although in the same experimental setup the reported IC_50_ of 167 nm was reasonably higher than for Ga-FAPI-04 (32 nm), the tumor accumulation in in HT1080FAP- and U87MG-xenografts was higher or equal while more persistent. Unfortunately, the tracer showed a dominant hepatobiliary elimination pathway and uptake in bones as well as in joints. Since the accumulation of a radiotracer in these areas would impair the imaging of the lower abdominal region and may lead to false positive findings in the skeleton, these were drawbacks our work tried to avoid.

While finalizing this manuscript, Dahl et al*.* introduced the automated radiolabeling of [^18^F]AlF-FAPI-74 on two different modules which enables a GMP compliant synthesis offering good yields and reproducibility (Dahl et al. 2021). Additionally Wang et al*.* published the clinical translation of [^18^F]AlF-FAPI-42 (termed ‘Al^18^F-NOTA-FAPI’) (Wang et al. 2021). In this case a hepatobiliary elimination together with an accumulation in the bone were observed in the U87MG- and A549-xenograft models. These drawbacks did not completely translate into the clinical application. This was addressed to be a result of the removal of gall bladder contents by food digestion in some patients as well as a lower FAP expression in human bone structure in general.

## Conclusion

In this study we have designed several ^18^F-labeled FAPI ligands based on our previously successful FAPI-scaffold. The preclinical investigation clearly demonstrated that [^18^F]AlF-FAPI-74 offers the most promising characteristics for clinical application. The novel ligand demonstrated good tumor uptake and retention. Owing to its rapid clearance from non-target tissue, PET imaging with [^18^F]AlF-FAPI-74 at early time points was superior to all other ligands investigated in this study. Finally, the clinical potential of the novel ligand could be demonstrated in a NSCLC cancer patient in a compassionate use scenario. In summary, [^18^F]AlF-FAPI-74 is an excellent and promising alternative to the well-known [^68^ Ga]Ga-FAPI-74 with the potential for large scale production and distribution by satellite concept.

## Supplementary Information

**Additional file 1.Fig S2:** Stability of 18F-labeled FAPI-74 in human serum. No degradation products were observed in the radio-HPLC traces at any given time point. Conditions: 0-30% acetonitrile in 10 minutes.

**Additional file 2.Fig S3:** PET scans (maximum intensity projections, acquisition at 40-60 min p.i.) of the discontinued radiotracers [18F]AlF-FAPI-42, -52, -72, -73, and -76. The white arrow indicates the site of the implanted tumor.

**Additional file 3.Fig S4:** Blocking the tumor uptake of 68Ga-labeled FAPI-74 by co-administration of 30 nmol unlabeled precursor. Shown are maximum intensity projections of the indicated time intervals. The white arrow indicates the site of the implanted tumor.

## Abbreviations

CAF: Cancer associated fibroblast
FAP: Fibroblast activating protein alpha
AlF: Aluminum fluoride complex
CD26 or DPP4: DIPEPTIDYL peptidase 4
PET: Positron emission tomography
PET/CT: Positron emission tomography / computer tomography
NSCLC: Non-small cell lung cancer
EC_50_: Half maximal effective concentration
NOTA: 1,4,7-Triazacyclononane-1,4,7-triacetic acid
FAPI: Fibroblast activating protein alpha inhibitor
KHCO_3_: Potassium bicarbonate
NaCl: Sodium chloride
NaOAc: Sodium acetate
AlCl_3_: Aluminum chloride
DMSO: Dimethyl sulfoxide
SPE: Solid-phase extraction
log D: Logarithmic value of distribution coefficient
cpm: Counts per minute
PBS: Phosphate buffered saline
DMEM: Dulbecco’s modified Eagle’s medium
FCS: Fetal calf serum
NaOH: Sodium hydroxide
SDS: Sodium dodecyl sulfate
glycine–HCl: Glycine hydrochloride
%AD: Percentage of applied dose
OSEM: Ordered subset expectation maximization algorithm
SUV: Standardized uptake value
SUVmax: Maximum SUV
SUVmean: Mean SUV
%ID/g: Percentage of injected dose per gram of tissue
p.i.: Post injection

## References

[CR1] Basuli F, Zhang X, Jagoda EM, Choyke PL, Swenson RE (2016). Facile room temperature synthesis of fluorine-18 labeled fluoronicotinic acid-2,3,5,6-tetrafluorophenyl ester without azeotropic drying of fluorine-18. Nucl Med Biol.

[CR2] Calais J (2020). FAP: the next billion dollar nuclear theranostics target?. J Nucl Med.

[CR3] Cardinale J, Schafer M, Benesova M, Bauder-Wust U, Leotta K, Eder M (2017). Preclinical evaluation of (18)F-PSMA-1007, a new prostate-specific membrane antigen ligand for prostate cancer imaging. J Nucl Med.

[CR4] Dahl K, Jussing E, Bylund L, Moein MM, Samén E, Tran T (2021). Fully automated production of the fibroblast activation protein radiotracer [18F]FAPI-74. J Lab Compd Radiopharm.

[CR5] Gascard P, Tlsty TD (2016). Carcinoma-associated fibroblasts: orchestrating the composition of malignancy. Genes Dev.

[CR6] Giesel FL, Kratochwil C, Lindner T, Marschalek MM, Loktev A, Lehnert W (2019). (68)Ga-FAPI PET/CT: biodistribution and preliminary dosimetry estimate of 2 DOTA-containing FAP-targeting agents in patients with various cancers. J Nucl Med.

[CR7] Giesel FL, Heussel CP, Lindner T, Rohrich M, Rathke H, Kauczor HU (2019). FAPI-PET/CT improves staging in a lung cancer patient with cerebral metastasis. Eur J Nucl Med Mol Imaging.

[CR8] Giesel FL, Knorr K, Spohn F, Will L, Maurer T, Flechsig P (2019). Detection efficacy of (18)F-PSMA-1007 PET/CT in 251 patients with biochemical recurrence of prostate cancer after radical prostatectomy. J Nucl Med.

[CR9] Giesel FL, Kratochwil C, Schlittenhardt J, Dendl K, Eiber M, Staudinger F (2021). Head-to-head intra-individual comparison of biodistribution and tumor uptake of 68Ga-FAPI and 18F-FDG PET/CT in cancer patients. Eur J Nucl Med Mol Imaging.

[CR10] Giesel F, Adeberg S, Syed M, Lindner T, Jimenez LD, Mavriopoulou E, et al. FAPI-74 PET/CT using either (18)F-AlF or Cold-kit (68)Ga-labeling: biodistribution, radiation dosimetry and tumor delineation in lung cancer patients. J Nucl Med. 2020. 10.2967/jnumed.120.245084 PMC8679591 32591493

[CR11] Jacobson O, Kiesewetter DO, Chen X (2015). Fluorine-18 radiochemistry, labeling strategies and synthetic routes. Bioconjug Chem.

[CR12] Jansen K, Heirbaut L, Cheng JD, Joossens J, Ryabtsova O, Cos P (2013). Selective inhibitors of fibroblast activation protein (FAP) with a (4-Quinolinoyl)-glycyl-2-cyanopyrrolidine Scaffold. ACS Med Chem Lett.

[CR13] Jansen K, Heirbaut L, Verkerk R, Cheng JD, Joossens J, Cos P (2014). Extended structure-activity relationship and pharmacokinetic investigation of (4-quinolinoyl)glycyl-2-cyanopyrrolidine inhibitors of fibroblast activation protein (FAP). J Med Chem.

[CR14] Jiang D, Chen X, You Z, Wang H, Zhang X, Li X,·et al,  (2021). Comparison of [68Ga]Ga-FAPI-04 and [18F]-FDG for the detection of primary and metastatic lesions in patients with gastric cancer: a bicentric retrospective study. Eur J Nucl Med Mol Imaging.

[CR15] Kratochwil C, Flechsig P, Lindner T, Abderrahim L, Altmann A, Mier W (2019). (68)Ga-FAPI PET/CT: tracer uptake in 28 different kinds of cancer. J Nucl Med.

[CR16] Kuten J, Levine C, Shamni O, Pelles S, Wolf I, Lahat G (2021). Head-to-head comparison of [68Ga]Ga-FAPI-04 and [18F]-FDG PET/CT in evaluating the extent of disease in gastric adenocarcinoma. Eur J Nucl Med Mol Imaging.

[CR17] Lamprecht S, Sigal-Batikoff I, Shany S, Abu-Freha N, Ling E, Delinasios GJ, et al. Teaming up for trouble: cancer cells, transforming growth factor-beta1 signaling and the epigenetic corruption of stromal naive fibroblasts. Cancers (Basel). 2018;10(3). 10.3390/cancers10030061 PMC5876636 29495500

[CR18] Lindner T, Loktev A, Altmann A, Giesel F, Kratochwil C, Debus J (2018). Development of quinoline-based theranostic ligands for the targeting of fibroblast activation protein. J Nucl Med.

[CR19] Lindner T, Loktev A, Giesel F, Kratochwil C, Altmann A, Haberkorn U (2019). Targeting of activated fibroblasts for imaging and therapy. EJNMMI Radiopharm Chem.

[CR20] Lindner T, Altmann A, Kraemer S, Kleist C, Loktev A, Kratochwil C, et al. Design and development of (99m)Tc labeled FAPI-tracers for SPECT-imaging and 188Re therapy. J Nucl Med. 2020. 10.2967/jnumed.119.239731 PMC7539653 32169911

[CR21] Loktev A, Lindner T, Mier W, Debus J, Altmann A, Jäger D (2018). A tumor-imaging method targeting cancer-associated fibroblasts. J Nucl Med.

[CR22] Loktev A, Lindner T, Burger EM, Altmann A, Giesel F, Kratochwil C, et al. Development of novel FAP-targeted radiotracers with improved tumor retention. J Nucl Med. 2019. 10.2967/jnumed.118.224469 PMC6785792 30850501

[CR23] Marsh T, Pietras K, McAllister SS (2013). Fibroblasts as architects of cancer pathogenesis. Biochim Biophys Acta.

[CR24] McBride WJ, Sharkey RM, Karacay H, D'Souza CA, Rossi EA, Laverman P (2009). A novel method of 18F radiolabeling for PET. J Nucl Med.

[CR25] Olberg DE, Arukwe JM, Grace D, Hjelstuen OK, Solbakken M, Kindberg GM (2010). One step radiosynthesis of 6-[(18)F]fluoronicotinic acid 2,3,5,6-tetrafluorophenyl ester ([(18)F]F-Py-TFP): a new prosthetic group for efficient labeling of biomolecules with fluorine-18. J Med Chem.

[CR26] Plava J, Cihova M, Burikova M, Matuskova M, Kucerova L, Miklikova S (2019). Recent advances in understanding tumor stroma-mediated chemoresistance in breast cancer. Mol Cancer.

[CR27] Pure E, Lo A (2016). Can targeting stroma pave the way to enhanced antitumor immunity and immunotherapy of solid tumors?. Cancer Immunol Res.

[CR28] Richarz R, Krapf P, Zarrad F, Urusova EA, Neumaier B, Zlatopolskiy BD (2014). Neither azeotropic drying, nor base nor other additives: a minimalist approach to (18)F-labeling. Org Biomol Chem.

[CR29] Richter S, Wuest F (2014). 18F-labeled peptides: the future is bright. Molecules.

[CR30] Toms J, Kogler J, Maschauer S, Daniel C, Schmidkonz C, Kuwert T (2020). Targeting fibroblast activation protein: radiosynthesis and preclinical evaluation of an (18)F-labeled FAP inhibitor. J Nucl Med.

[CR31] Wang S, Zhou X, Xu X, Ding J, Liu S, Hou X, et al. Clinical translational evaluation of Al18F-NOTA-FAPI for fibroblast activation protein-targeted tumour imaging. Eur J Nucl Med Mol Imaging. 2021. 10.1007/s00259-021-05470-5 34165601

