# Supplementary material for: 18F-labeled tracers targeting fibroblast activation protein
Source: EJNMMI Radiopharm Chem. 2021 Aug 21;6:26. doi: 10.1186/s41181-021-00144-x (PMC8380212; doi:10.1186/s41181-021-00144-x)
Supplement: Supplementary file 1 — Additional file 1.Fig S2: Stability of 18F-labeled FAPI-74 in human serum. No degradation products were observed in the radio-HPLC traces at any given time point. Conditions: 0-30% acetonitrile in 10 minutes. [file 41181_2021_144_MOESM1_ESM.docx]

**Supporting Information**

**Additional materials and detailed compound synthesis p. 1-4**

**Compound analysis p. 4-5**

**Supplemental figures and table p. 5-8**

**Additional references p. 8**

**Reagents**

All solvents and non-radioactive reagents were obtained in reagent grade from ABCR (Karlsruhe, Germany), Sigma-Aldrich (München, Germany), Acros Organics (Geel, Belgium) or VWR (Bruchsal, Germany) and were used without further purification. NOTA (2,2′,2”-(1,4,7-triazacyclononane-1,4,7-triyl)triacetic acid) and NODAGA NHS-ester (2,2′-(7-(1-carboxy-4-((2,5-dioxopyrrolidin-1-yl)oxy)-4-oxobutyl)-1,4,7-triazacyclononane-1,4-diyl)diacetic acid) were obtained from CheMatech (Dijon, France). The 4-nitrophenyl ester of NOTA was prepared following the analogous protocol for DOTA (*1*). (*S*)-*N*-(2-(2-cyano-4,4-difluoropyrrolidin-1-yl)-2-oxoethyl)-6-(3-(4-*tert*-butoxycarbonylpiperazin-1-yl)-1-propoxy)quinoline-4-carboxamide and(*S*)-*N*-(2-(2-cyanopyrrolidin-1-yl)-2-oxoethyl)-6-(3-(4-*tert*-butoxycarbonylpiperazin-1-yl)-1-propoxy)quinoline-4-carboxamide were synthesized as already described.(*2,3*)

**Compound Synthesis**

**

**

**Supplemental figure 1** depicts the synthesis of FAPI-73 and -75 as well as the route to the final radiotracers.

FAPI-42


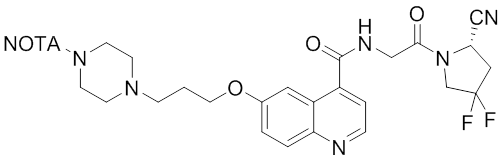


1.65 mg (2.81 µmol) of (*S*)-*N*-(2-(2-cyano-4,4-difluoropyrrolidin-1-yl)-2-oxoethyl)-6-(3-(4-*tert*-butoxycarbonylpiperazin-1-yl)-1-propoxy)quinoline-4-carboxamide were dissolved in 150 µL of a 2:1-mixture of trifluoroacetic acid and acetonitrile. The solvents were removed by vacuum evaporation, the residue triturated with diethyl ether and dried. A solution of 2.51 mg (5.92 µmol) of NOTA-*p*-nitrophenol in 100 µL dimethyl sulfoxide followed by 5.00 µL (3.65 mg; 36.1 µmol) triethylamine were added to the precipitate and reacted for 120 minutes. After checking for completeness, the mixture was diluted with 1 mL of 25% acetonitrile in water and subjected to HPLC purification. 1.91 mg (2.47 µmol; 88%) of the title compound were obtained after freeze-drying.

**LC-MS** R_t_ 9.37 min, m/z 386.6807 [M+2H]^2+^ calc: 386.6831

FAPI-52


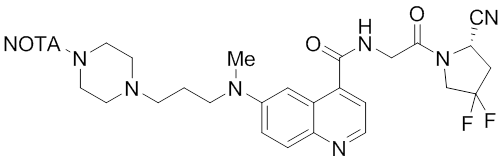


1.12 mg (1.43 µmol; 94%) were obtained following the protocol described for FAPI-42.

**LC-MS** R_t_ 9.42 min, m/z 393.1957 [M+2H]^2+^ calc: 393.1989

FAPI-72


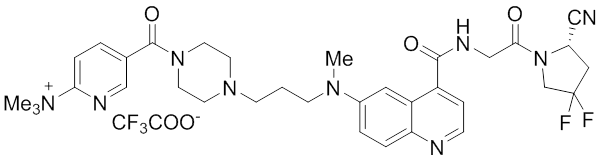


9.80 mg (12.6 µmol; 70%) were obtained following the protocol described for FAPI-73.

**LC-MS** R_t_ 9.37 min, m/z 662.3237 [M-CF_3_CO_2_]^+^ calc: 662.3373

FAPI-73


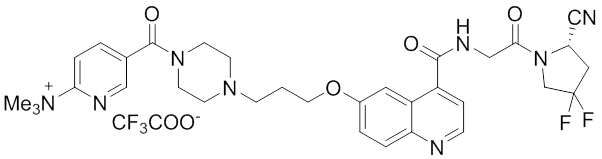


10.95 mg (18.7 µmol) of (*S*)-*N*-(2-(2-cyano-4,4-difluoropyrrolidin-1-yl)-2-oxoethyl)-6-(3-(4-*tert*-butoxycarbonylpiperazin-1-yl)-1-propoxy)quinoline-4-carboxamide were deprotected for 30 min by 100 µL acetonitrile and 200 µL trifluoroacetic acid. After removal of the solvents by vacuum evaporation and washing with diethyl ether 15.02 mg (9.27 µmol) *N*,*N*,*N*-trimethyl-5-((2,3,5,6-tetrafluorophenoxy)-carbonyl)pyridine-2-aminium chloride was added and the mixture dissolved in 200 µL dimethylformamide and 10.0 µL (7.30 mg; 72.3 µmol) triethylamine. After 120 min the mixture was purified by HPLC and 11.24 mg (14.7 µmol; 79%) of the title compound were obtained freeze-drying.

**LC-MS** R_t_ 9.28 min, m/z 649.2892 [M-CF_3_CO_2_]^+^ calc: 649.3075

FAPI-74


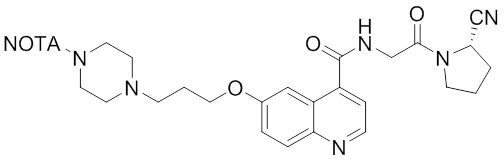


3.03 mg (4.12 µmol; 77%) of the title compound were obtained from 2.95 mg (5.35 µmol) (*S*)-*N*-(2-(2-cyanopyrrolidin-1-yl)-2-oxoethyl)-6-(3-(4-*tert*-butoxycarbonylpiperazin-1-yl)-1-propoxy)quinoline-4-carboxamide was used as starting material following the protocol described for FAPI-42.

**1H-NMR** (500 MHz, D_2_O): 8.77 (d), 8.03 (d), 7.58 (dd), 7.52 (dd), 4.82 (t), 4.36-4.30 (m), 3.81-3.54 (m), 3.30-2.78 (m), 2.35-2.20 (m); **LC-MS** R_t_ 8.83 min, m/z 736.3584 [M+H]^+^ calc: 736.3777

(*S*)-*N*-(2-(2-cyano-4,4-difluoropyrrolidin-1-yl)-2-oxoethyl)-6-(3-(4-(γ,γ-di-*tert*-butyl)-l-carboxy-glutamylpiperazin-1-yl)-1-propoxy)quinoline-4-carboxamide


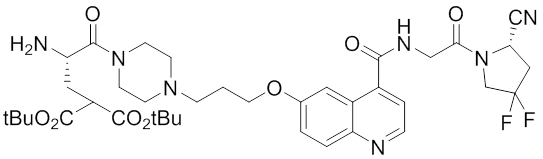


14.04 mg (23.9 µmol) of (*S*)-*N*-(2-(2-cyano-4,4-difluoropyrrolidin-1-yl)-2-oxoethyl)-6-(3-(1-*tert*-butoxycarbonyl-piperidin-4-yl)-1-propoxy)quinoline-4-carboxamide were dissolved in 50 µL acetonitrile and 100 µL trifluoroacetic acid. After 10 min the volatiles were removed by vacuum evaporation and the residue was washed with diethyl ether. A solution of 14.95 mg (28.4 µmol) Fmoc-l-Gla(tBu)_2_-OH, 7.74 mg (57.4 µmol) HOBt, 13.46 mg (35.5 µmol) HBTU and 20.0 µL (14.8 mg; 115 µmol) DIPEA in 200 µL dimethylformamide was added to the dried residue. After 60 min 50.0 µL (50.4 mg; 578 µmol) morpholine were added and the product was isolated by HPLC after 30 min. 15.95 mg (20.7 µmol; 86%) of the title compound were obtained after freeze drying.

**LC-MS** R_t_ 12.85 min, m/z 772.3643 [M+H]^+^ calc: 772.3840

FAPI-75


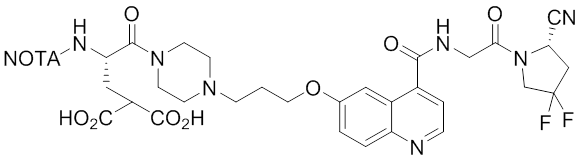


3.37 mg (4.37 µmol) of (*S*)-*N*-(2-(2-cyano-4,4-difluoropyrrolidin-1-yl)-2-oxoethyl)-6-(3-(4-(γ,γ-di-*tert*-butyl)-l-carboxyglutamylpiperazin-1-yl)-1-propoxy)quinoline-4-carboxamide and 4.52 mg (10.7 µmol) NOTA-*p*-nitrophenol were dissolved in 100 µL dimethylformamide and 10.0 µL (7.30 mg; 72.3 µmol) triethylamine. After HPLC-purification and freeze-drying the intermediate compound was deprotected by incubation in a solution of 50 µL acetonitrile, 100 µL trifluoroacetic acid, 2.5 µL triisopropylsilane and 2.5 µL water. 2.62 mg (2.77 µmol; 63%) were obtained after HPLC-purification and freeze-drying.

**LC-MS** R_t_ 9.54 min, m/z 451.1928 [M+2H]^2+^ calc: 473.1993

FAPI-76


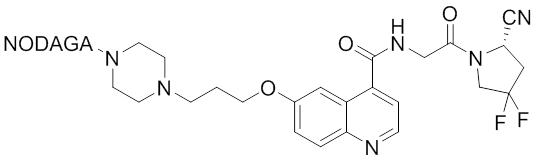


5.56 mg (6.59 µmol; 70%) were obtained following the protocol described for FAPI-74 using NODAGA NHS-ester instead of the 4-nitrophenyl ester of NOTA.

**LC-MS** R_t_ 9.22 min, m/z 897.2665 [M+Fe-2H]^+^ calc: 897.2914

**Compound Analysis**

Reverse-phase high-performance liquid chromatography (RP-HPLC) was conducted using linear gradients of acetonitrile in water (0-100% acetonitrile in 5 min; 0.1% TFA; flowrate 2 mL/min) on a Chromolith Performance RP-18e column (100 × 3 mm; Merck KGaA Darmstadt, Germany). UV-absorbance was detected at 214 nm. An additional γ-detector was used for the HPLC-analysis of radioactive compounds. HPLC-MS characterization was performed on an ESI mass spectrometer (Exactive, Thermo Fisher Scientific, Waltham, MA, USA) connected to an Agilent 1200 HPLC system with a Hypersil Gold C18 1.9 μm column (200 × 2.1 mm; 0-100% acetonitrile in 20 min; flowrate 200 μL/min). Analytical Radio-HPLC was performed using a Chromolith Performance RP-18e column (100×3mm; Merck; 0-100% acetonitrile in 5 min; flowrate 2 mL/min). HPLC-purifications were performed on a LaPrep P110-System (Knauer, Berlin, Germany) and a Reprosil Pur 120 column (C18-aq 5 μm 250 × 25mm; Dr. Maisch, Ammerbuch-Entringen, Germany). The water/acetonitrile-gradient (15 or 25 min; 0.1% TFA; flowrate 20 mL/min) was modified for the individual products. The purity of isolated compounds was checked by means of LCMS. Purifications by means of Radio-HPLC was performed using a Chromolith Performance RP-18e column (100×4.6mm; Merck; 0-40% acetonitrile in 10 min; flowrate 2 mL/min). The 1H-NMR spectra of FAPI-74 was recorded using a Bruker Avance III 500 MHz spectrometer (Bruker BioSpin, Rheinstetten, Germany) at the IPMB Heidelberg, Germany.

**
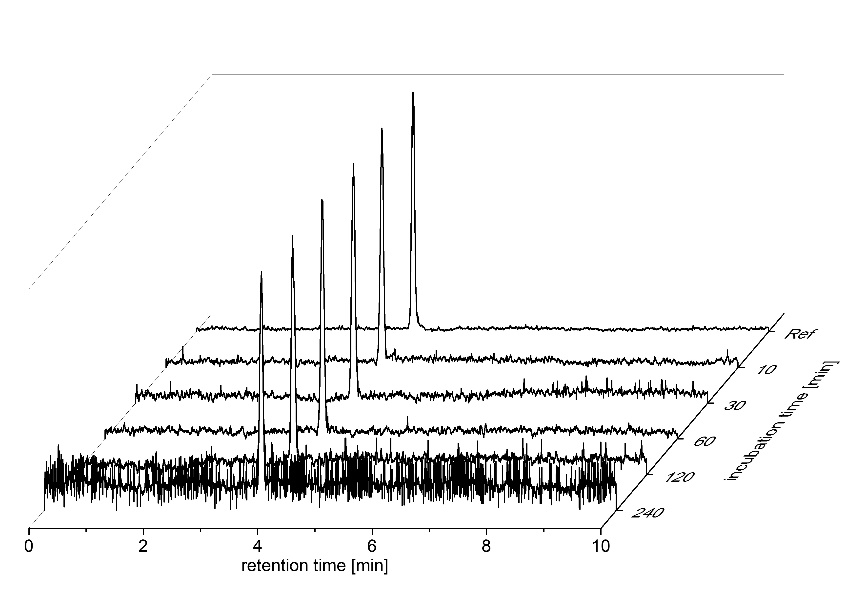
**

**SUPPLEMENTAL FIGURE 2:** Stability of ^18^F-labeled FAPI-74 in human serum. No degradation products were observed in the radio-HPLC traces at any given time point. Conditions: 0-30% acetonitrile in 10 minutes.

**
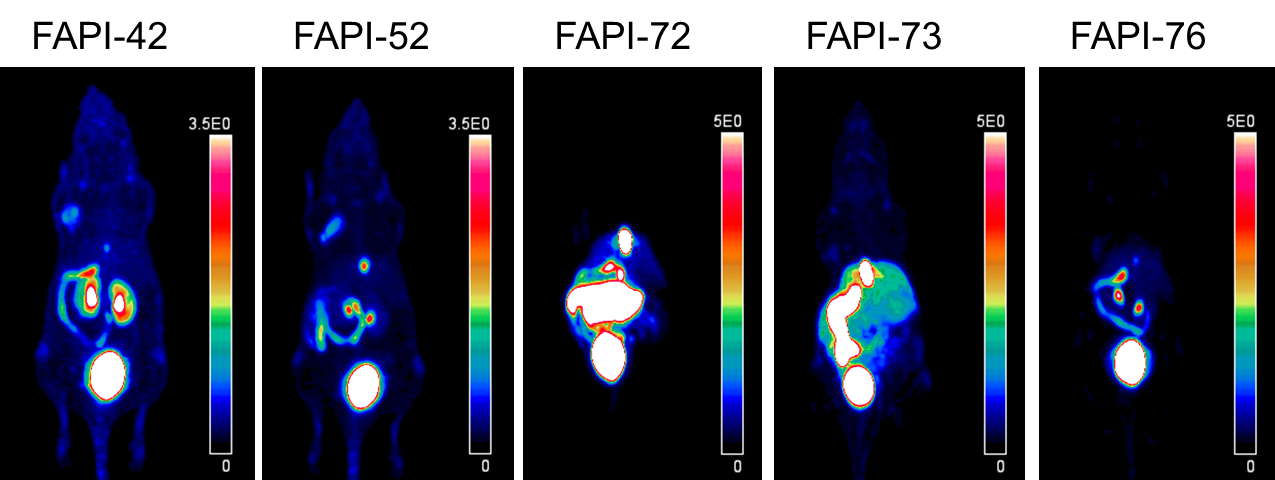
**

**SUPPLEMENTAL FIGURE 3:** PET scans (maximum intensity projections, acquisition at 40-60 min p.i.) of the discontinued radiotracers [^18^F]AlF-FAPI-42, -52, -72, -73, and -76. The white arrow indicates the site of the implanted tumor.

|  |  | **Tumor Uptake** | **(SUVmean)** |  |
| --- | --- | --- | --- | --- |
| **FAPI** | **8 min** | **22 min** | **57 min** | **120 min** |
| -42 | 1.05 | 1.0 | 0.73 | 0.60 |
| -52 | 0.65 | 0.55 | 0.47 | 0.20 |
| -72 | 0.19 | 0.15 | 0.13 | 0.1 |
| -73 | 0.45 | 0.44 | 0.42 | 0.33 |
| -74 | 1.58 | 1.81 | 1.91 | 1.3 |
| -75 | 1.55 | 2.06 | 2.24 | 1.9 |
| -76 | 0.38 | 0.29 | 0.27 | 0.12 |

**SUPPLEMENTAL TABLE 1:** Tumor-SUVmean values of all mentioned ^18^F labeled FAPI-derivatives in HT1080-FAP transplanted nude mice at different time points. The time points 8 min, 22 min and 57 min were selected from the applied 28-frame 3D-OSEM+MAP (Siemens) reconstruction method.

**
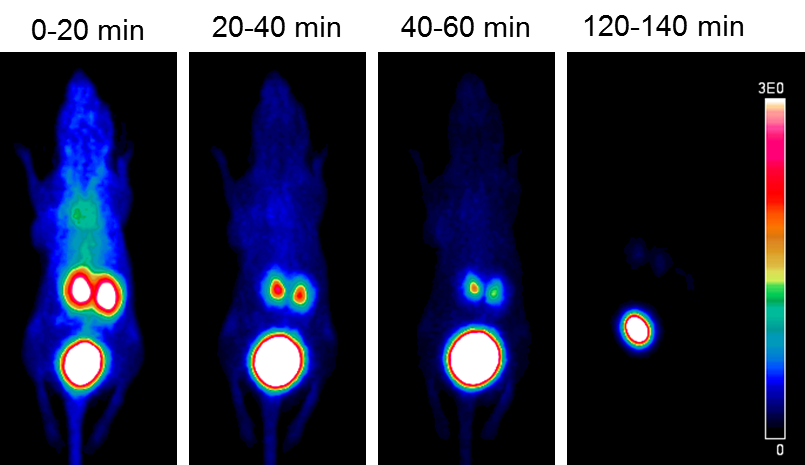
**

**SUPPLEMENTAL FIGURE 4:** Blocking the tumor uptake of ^68^Ga-labeled FAPI-74 by co-administration of 30 nmol unlabeled precursor. Shown are maximum intensity projections of the indicated time intervals. The white arrow indicates the site of the implanted tumor.

**REFERENCES:**

**1.** Mier W, Hoffend J, Kramer S, et al. Conjugation of DOTA using isolated phenolic active esters: the labeling and biodistribution of albumin as blood pool marker. *Bioconjug Chem.* 2005;16:237-240.

**2.** Lindner T, Loktev A, Altmann A, et al. Development of Quinoline-Based Theranostic Ligands for the Targeting of Fibroblast Activation Protein. *J Nucl Med.* 2018;59:1415-1422.

**3.** Loktev A, Lindner T, Mier W, et al. A Tumor-Imaging Method Targeting Cancer-Associated Fibroblasts. *J Nucl Med.* 2018;59:1423-1429.
