# Supplementary figures and images for: 18F-labeled tracers targeting fibroblast activation protein
Source: EJNMMI Radiopharm Chem. 2021 Aug 21;6:26. doi: 10.1186/s41181-021-00144-x (PMC8380212; doi:10.1186/s41181-021-00144-x)

## Slide 1
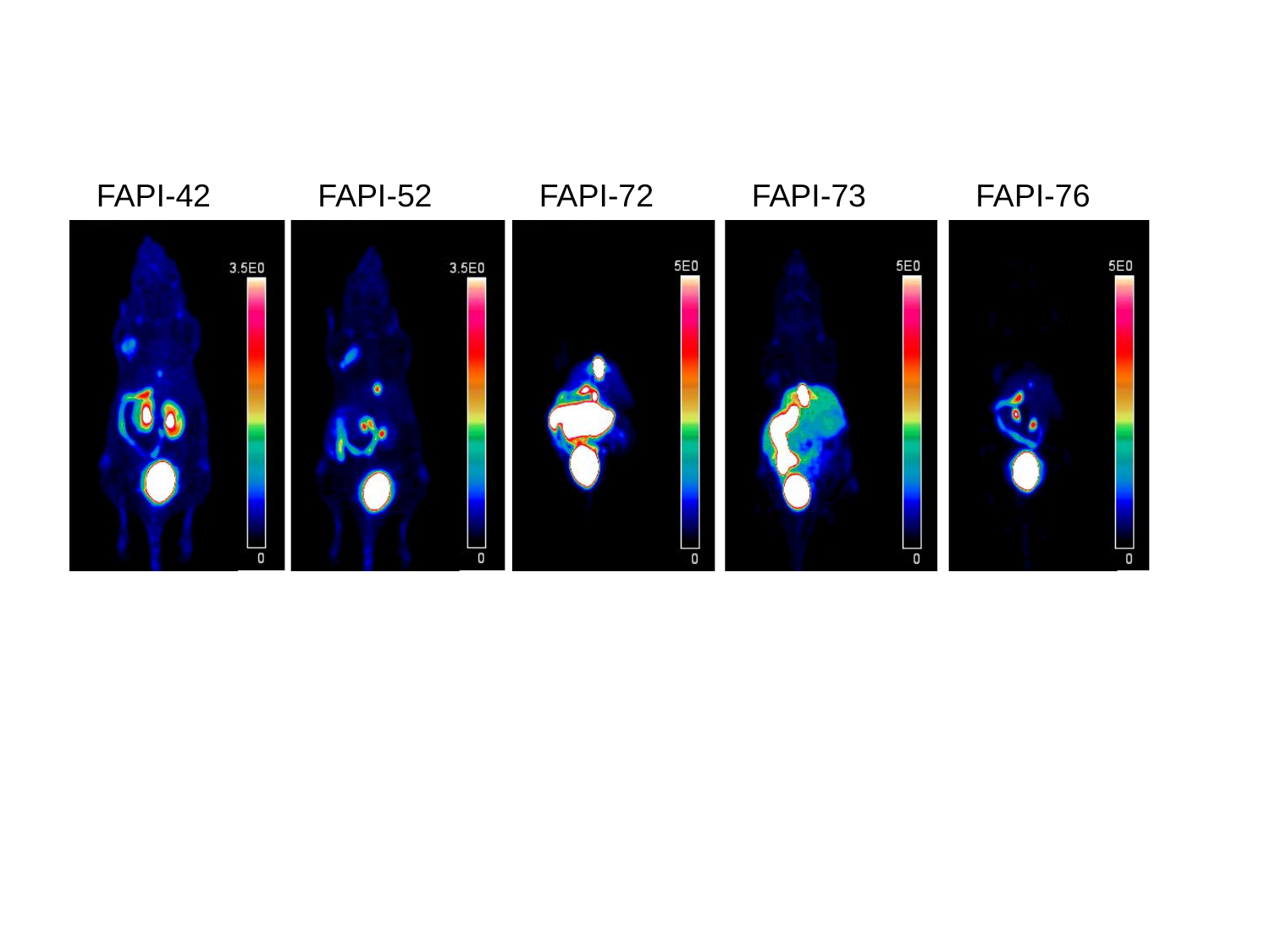

FAPI-42
FAPI-52
FAPI-72
FAPI-73
FAPI-76

Supplement: Supplementary file 2 — Additional file 2.Fig S3: PET scans (maximum intensity projections, acquisition at 40-60 min p.i.) of the discontinued radiotracers [18F]AlF-FAPI-42, -52, -72, -73, and -76. The white arrow indicates the site of the implanted tumor. [file 41181_2021_144_MOESM2_ESM.pptx]

## Slide 1
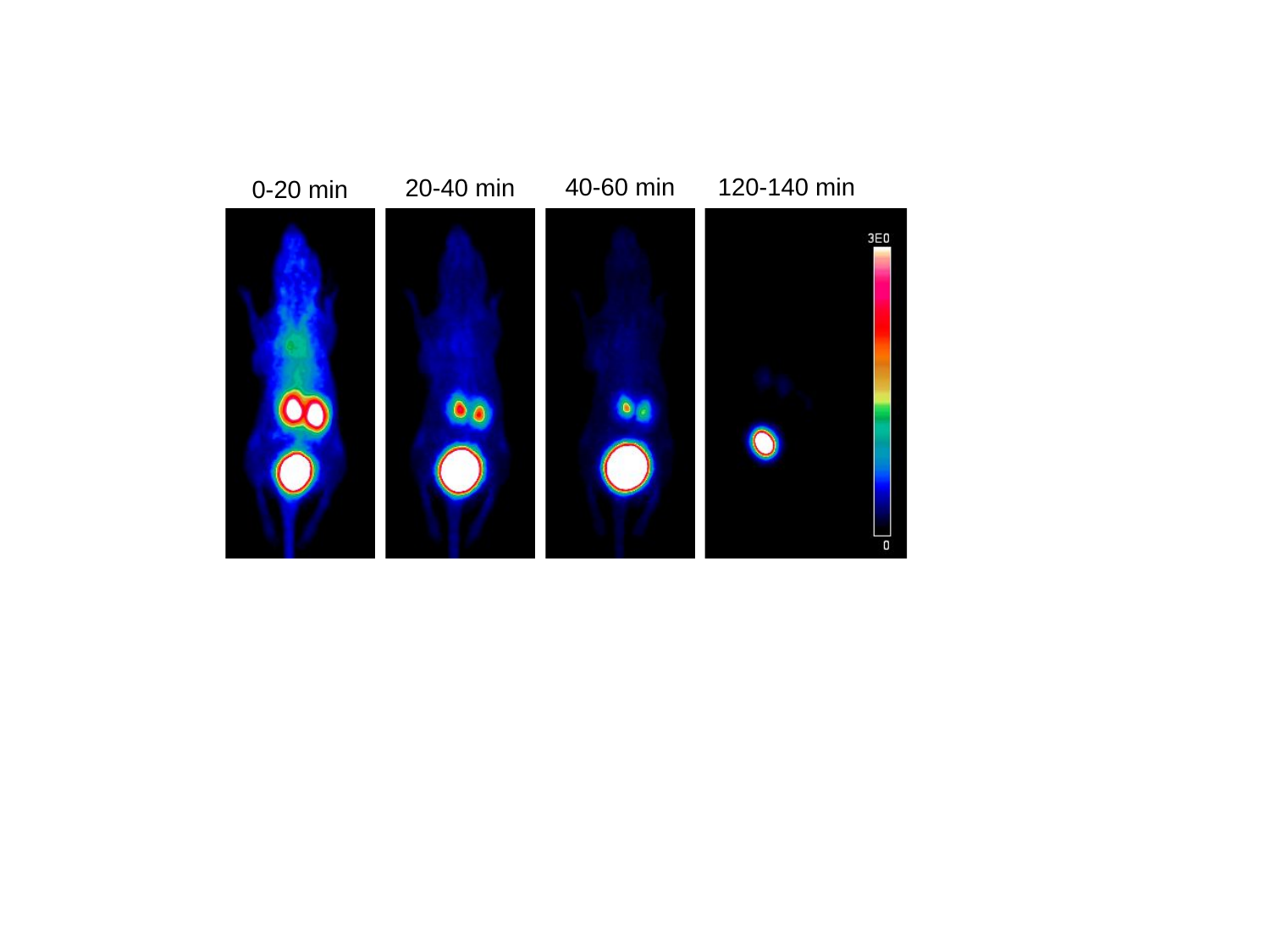

120-140 min
40-60 min
20-40 min
0-20 min

Supplement: Supplementary file 3 — Additional file 3.Fig S4: Blocking the tumor uptake of 68Ga-labeled FAPI-74 by co-administration of 30 nmol unlabeled precursor. Shown are maximum intensity projections of the indicated time intervals. The white arrow indicates the site of the implanted tumor. [file 41181_2021_144_MOESM3_ESM.pptx]
